# 

**Published:** 1898-10

**Authors:** 


☞ During October bills for subscription will be mailed to every subscriber who is in arears, and we must insist on our friends *dividing* with us at least. This is collection season. "Installments" will be accepted if not convenient to remit in full.

